# There Is a Limit to Your Openness: Mental Illness Stigma Mediates Effects of Individual Traits on Preference for Psychiatry Specialty

**DOI:** 10.3389/fpsyt.2019.00775

**Published:** 2019-10-30

**Authors:** Edita Fino, Alessandro Agostini, Michela Mazzetti, Valentina Colonnello, Elisa Caponera, Paolo Maria Russo

**Affiliations:** ^1^Department of Experimental, Diagnostic and Specialty Medicine (DIMES), Alma Mater Studiorum—University of Bologna, Bologna, Italy; ^2^National Institute for the Educational Evaluation of Instruction and Training (INVALSI), Rome, Italy

**Keywords:** mental illness stigma, personality traits, openness to experience, psychiatry specialty, medical students

## Abstract

**Objective:** The widening gap between the need for mental health professionals and the low percentages of medical students pursuing a psychiatric career urges an examination of how individual traits, stigma attitudes, and related intended behaviors interact to better explain the variance in preferences for psychiatry as a specialty choice.

**Methods:** Participants were second-year, preclinical medical students at Bologna University, Italy. The study consisted in completion of an online questionnaire evaluating preferences for the psychiatry specialty (one single item and a scenario-based response), personality traits (the Big Five Questionnaire), attitudes (Mental Illness for Clinicians’ Attitude scale), behaviors (Reported and Intended Behavior Scale), and fears toward mental illness (questionnaire created *ad hoc*). Sociodemographic data were also collected.

**Results:** A total of 284 medical students [58.8% female, mean (SD) age 20.47 ± 1.90] completed the questionnaire. Preference for the psychiatry specialty was significantly and positively associated with openness to experience and negatively related with Mental Illness for Clinicians’ Attitude scale and Reported and Intended Behavior Scale. The full-mediation model provided good indices explaining 18% of the variance. Mental illness stigma was strongly and negatively associated with both openness to experience and preference for psychiatry, and the mediation results evidenced a positive and significant effect.

**Conclusions:** Mental illness stigma influences medical students’ choice of psychiatry as a specialty, accounting for the effects of the openness to experience trait. Stigma awareness and reduction programs should be introduced as early as possible in medical education.

## Introduction

The psychiatry specialty remains an unpopular choice for medical students (1, 2) despite the soaring demand for mental health professionals worldwide (3). Marked by significant differences in clinical environment, physician responsibility, and patient exposure, specific disciplines within the medical profession are believed to require and to typically attract specific individual characteristics (4–6). Psychiatry is traditionally clustered in this body of research as a stand-alone choice compared to other medical specialties (e.g., internal medicine and surgery) based on evidence suggesting that psychiatrists might have distinct characteristics in their personality profiles (4, 7–9). Indeed, among other individual traits, openness to experience is the most reliably reported in psychiatrists and medical students with a predilection for psychiatry as a future career choice (4, 10–14), but see (9) for mixed results. Yet, perhaps because it is seemingly self-evident, how openness to experience affects medical students’ choice of psychiatry specialty has not been systematically examined. Because persons who score higher on openness are tendentially more open minded and people oriented, they might be more intellectually curious toward the worldview of individuals with mental illness and, hence, more attracted toward psychiatry as a specialty choice (15). By virtue of the same tendency, high-openness individuals might experience fewer barriers to or fear from a close-contact experience with patients affected by mental illness and might exhibit lower levels of prejudice related to mental illness. Considering that prejudice can be reliably related to some personality traits more than others (16–18) and, therefore, it might be tendentially less present in those projecting themselves in a psychiatric career, related research has highlighted how stigmatizing attitudes about persons affected by mental illness may influence medical students’ view and choice of psychiatry as a medical specialty (19).

While there are differing views with regard to the theoretical conceptualization of mental illness stigma, there is general agreement that it is a multifaceted concept including stereotypical beliefs and negative attitudes (i.e., prejudice) as well as behavioral tendencies (i.e., discrimination) usually expressed through avoidance or social distance (20–25). Some works (20, 21) have highlighted that prejudiced persons not only endorse negative stereotypes and attitudes, but they also have negative emotional reactions toward persons with mental health conditions. For instance, fear is suggested as an important factor underlying negative attitudes toward people with mental illness leading to avoidant and discriminatory behavior (25).

The pervasive nature of stigma is such that stigmatization also extends to those working in mental health professions in general and to psychiatrists in particular, including psychiatry as both an academic and a practical discipline. Research shows that, within the medical profession, psychiatry is viewed as lacking scientific basis and as not being medical enough (2, 26, 27). Increasingly healthcare professionals, including doctors and medical students, are identified as significant sources of prejudice and discrimination (19, 28–33). Medical students in particular likely view people with mental illnesses as unpredictable, dangerous, and untreatable, while they express distancing attitudes toward them (34–36). The stigma of psychiatry and psychiatrists negatively affects mental health care provision not only *via* reduced patients’ help-seeking behavior and compliance but primarily through difficulties in recruiting young psychiatrists from among medical graduates (1, 2). The core notion backed by this body of research is that stereotypical attitudes about mental illness may play an essential role in tainting the image of psychiatry as an attractive career choice, and whether such influence is independent from and/or might outweigh the role of personality characteristics traditionally associated with a preference for psychiatry (i.e., openness to experience) is a question that warrants examination. In fact, the openness to experience trait does not preclude one from holding stigmatizing views of mental illness, just as being a psychiatrist by profession does not automatically mean one is immune to stereotypes toward patients with mental illness (33, 37). However, as far as we are aware, no study has examined whether and to what extent the relationship between an openness to experience and the choice of psychiatry as a future medical career is influenced by stereotypical attitudes and behaviors toward persons affected by mental illness. Understanding the interaction between individual traits and mental illness stigma can shed light on the variance in medical students’ preferences for psychiatry as a specialty. Such an investigation may be important especially for helping students question their own possibly stigmatizing attitudes toward people with mental illness and how these might influence their career choices. It may also be important, from an educational perspective, in order to sensibly address this issue in education programs and to establish stigma reduction initiatives in schools and universities.

Thus, the present study aims to evaluate whether and to what extent a preference for psychiatry as a specialty is associated with a higher openness to experience and with lower levels of mental illness stigma. Specifically, we examine the role of mental illness stigma in mediating the relationship between preference for psychiatry and the openness to experience trait by testing two (full vs. partial) mediation models. Based on the literature cited above, we hypothesized that mental illness stigma would outweigh the role that openness to experience trait plays in the preference for psychiatry by fully mediating that relationship.

## Materials and Methods

### Participants

The target sample included 284 second-year medical students [58.8% female, mean (SD) age: 20.47 (1.90)] at the University of Bologna, Italy, at the time of data collection. The study design was cross-sectional, and an online web survey was employed to collect data on medical career choice, personality traits, and stigmatizing attitudes, behaviors, and fears toward mental illness, as well as sociodemographic information. Participants were recruited through a psychology research website and received course credit for their participation. Response rates were 93%, and data collection took place from September to December 2018.

### Measures

#### Preference for the Psychiatry Specialty (One Single Item and a Scenario-Based Response)

**Figure 1:**
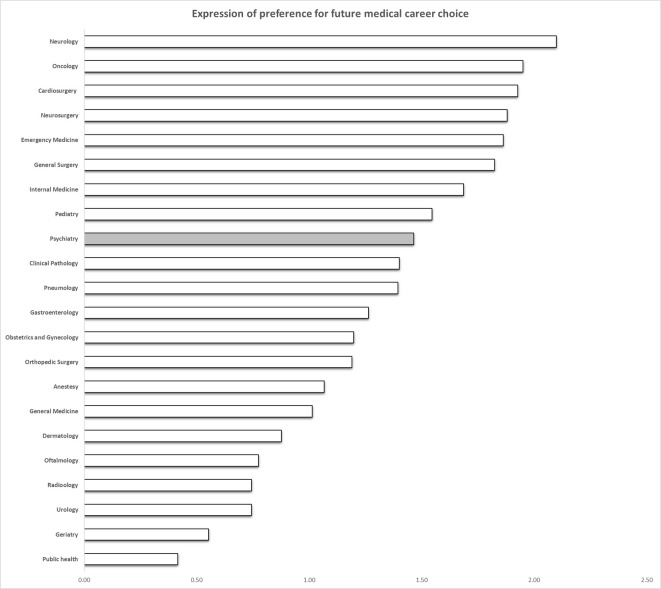
Bars indicate preferences for future career choice among medical specialties (range 1–5). The gray bar indicates preference for the psychiatry specialty.

Preference for the psychiatry specialty was assessed through two indicators: Participants rated their interest in the psychiatry specialty among 22 medical specialties (see **Figure 1**), using a 5-point Likert scale (1 = not at all interested to 5 = very interested). In addition, they completed the Scenario 2 of the Medical Situations Questionnaire (38), one of the most widely used measures of future specialty choice in a medical career (8). The scenario describes the case and treatment of a psychiatric patient and prompts students to imagine themselves in that situation and to evaluate related aspects of the medical practice. Participants had to imagine being a resident in a psychiatry clinic and to indicate their willingness to be in that situation by expressing their preference (1 = ”No,” 2 = ”indifferent,” and 3 = ”Yes”). The scenario was described as shown below:

“*You are working in the clinic and you see a distressed, 21-year old homeless man who appears to be nervous and describes hearing voices. You ask him about his family, his health and the medication he is taking. Afterwards you admit him in the hospital and make sure he receives appropriate treatment. Then you contact the social workers to arrange for his discharge and subsequent follow up in a sheltered accommodation. You see him regularly for therapy*.”

#### Openness to Experience (12 Items)

Openness to experience along with other personality traits was assessed through the Big Five Questionnaire (39), which is one of the most widely used measures of the Big Five dimensions and is aligned with other Big Five measures (40). The short form of the Big Five Questionnaire was used in the present study. It consisted of 60 items, with 12 items for each of the following dimensions: (1) Openness to experience pertains to the propensity for nurturing a wide variety of interests, being imaginative and insightful (Cronbach’s alpha = 0.73); (2) Conscientiousness pertains to self-regulation in both proactive and inhibitory modes (Cronbach’s alpha = 0.80); (3) Energy/Extraversion comprises activity, assertiveness, and self-confidence (Cronbach’s alpha = 0.72); (4) Agreeableness includes sympathy, kindness, and sensitivity toward others (Cronbach’s alpha = 0.82); and (5) Emotional Stability pertains to the ability to cope with/control anxiety and emotions (Cronbach’s alpha = 0.73). Respondents were asked to rate the degree to which each item described them on a 5-point scale (1 = very false for me; 5 = very true for me).

#### MICA-2 (16 Items)

Attitudes toward people with mental illness and toward psychiatry as a medical specialty were assessed through the adapted version of the Mental Illness Clinicians’ Attitude scale [MICA-2 (41)]. The adaptation was based on a translation and back translation of the scale by independent translators. MICA-2 is a single-factor scale composed of 16 items, with responses scored on a 6-point Likert scale (1 = strongly agree; 6 = strongly disagree). It yields a total score ranging from 16 to 96, with higher levels denoting more negative attitudes. The overall internal consistency of the scale in this study, based on Cronbach’s alpha, was 0.76, similar to that found in other studies using the original version with similar samples (41, 42).

#### RIBS (8 Items)

Intended behavior toward people with mental health problems was assessed through the Reported and Intended Behavior Scale [RIBS (43, 44)]. The RIBS is composed of eight items that come in two groups of four: the first four items (1–4) focus on previous experiences with people affected by mental illness (i.e., lived with, worked with, lived nearby, or had a relationship with a person with a mental health problem); the second group (5–8) focuses on future intentions to establish contact with people affected by mental health problems (i.e., live with, work with, live nearby, or have a relationship with a person with mental illness). Because the first group of items only report the prevalence of behaviors that respondents may or may not have had in the past, no final scale points are given for those. Only the second part of the scale (items 5–8) is used for computing a total RIBS score (35, 45). For the purpose of our study, we thus used items 5–8, concerning intended behavior, which were scored on a 5-point Likert scale (from 1 = ”strongly disagree to engage in the stated behavior” to 5 = ”strongly agree”) to calculate a total RIBS score, with higher levels indicating a higher willingness to engage in the target behavior. The overall internal consistency, based on Cronbach’s alpha among items 5–8 in this study, was 0.83, which is in line with previous studies involving the general population and medical students (35, 45). Item 1 of the RIBS scale was also assessed as a relevant variable to control for past exposure to and experience with persons affected by a mental condition: participants reported whether they currently had or had had in the past a close person with a mental health problem (scored as 1 = ”yes,” 2 = ”no,” 3 = ”don’t know”).

#### Fear of Mental Illness (11 Items)

Fear toward mental illness was measured through an *ad hoc* questionnaire evaluating the extent of fear that specific mental illnesses triggered in participants (see **Table 1**). Responses were scored on a 5-point Likert scale, from 1 = ”not at all” to 5 = ”very much,” for each mental disorder. The total score was calculated by adding together each single item. Higher scores indicated higher levels of mental illness fear. Items for specific mental illness fears were strongly interrelated, with an overall Cronbach’s alpha of 0.88.

**Table 1:** Means (SD) scores obtained in all scales for the entire sample and for male and female students separately.

	Total sample (*n* = 284)	Male students (n = 117)	Female students (*n* = 167)	*p*
Age	20.47 (1.90)	20.52 (2.66)	20.43 (1.11)	
**The Big Five**				
Openness to experience	3.63 (0.53)	3.68 (0.57)	3.60 (0.49)	0.237
Consciensiousness	4.00 (0.54)	3.87 (0.53)	4.08 (0.53)	0.001
Energy	3.13 (0.55)	3.11 (0.62)	3.15 (0.50)	0.643
Agreableness	3.56 (0.51)	3.50 (0.57)	3.60 (0.46)	0.148
Emotional Stability	3.00 (0.62)	3.17 (0.68)	2.88 (0.54)	<0.001
**Preference for Psychiatry**	1.46 (1.11)	1.46 (1.14)	1.47 (1.10)	0.876
**Mental Illness Stigma**				
MICA-2	43.02 (7.56)	44.95 (7.78)	41.68 (7.12)	0.001
RIBS	11.17 (3.49)	11.44 (3.69)	10.97 (3.34)	0.379
Personally knows someone with mental illness	1.80 (0.69)	1.85 (0.72)	1.77 (0.67)	0.387
**Fear of Mental Illness** (range 0–4)				
Schizophrenia	2.45 (1.12)	2.32 (1.18)	2.54 (1.07)	0.175
Depression	2.37 (1.15)	2.40 (1.20)	2.35 (1.12)	0.530
Addiction	2.15 (1.27)	1.99 (1.26)	2.27 (1.26)	0.228
OCD	1.90 (1.26)	1.74 (1.28)	2.01 (1.23)	0.068
Anxiety	1.87 (1.17)	2.32 (1.18)	1.98 (1.19)	0.094
PTSD	1.80 (1.05)	1.62 (0.97)	1.93 (1.09)	0.023
Burnout	1.77 (1.81)	1.68 (1.12)	1.83 (1.22)	0.245
BDD	1.70 (1.18)	1.70 (1.24)	1.69 (1.14)	0.778
Eating Disorder	1.64 (1.28)	1.36 (1.20)	1.84 (1.31)	0.001
Somatization	1.47 (1.04)	1.46 (1.12)	1.48 (0.93)	0.934
Hypocondria	1.14 (1.20)	1.11 (0.98)	1.16 (1.04)	0.617

*PTSD, posttraumatic stress disorder; BDD, body dysmorphic disorder; OCD, obsessive compulsive disorder.*

Level of statistical significance set at p < 0.01.

### Statistical Analysis

**Figure 2:**
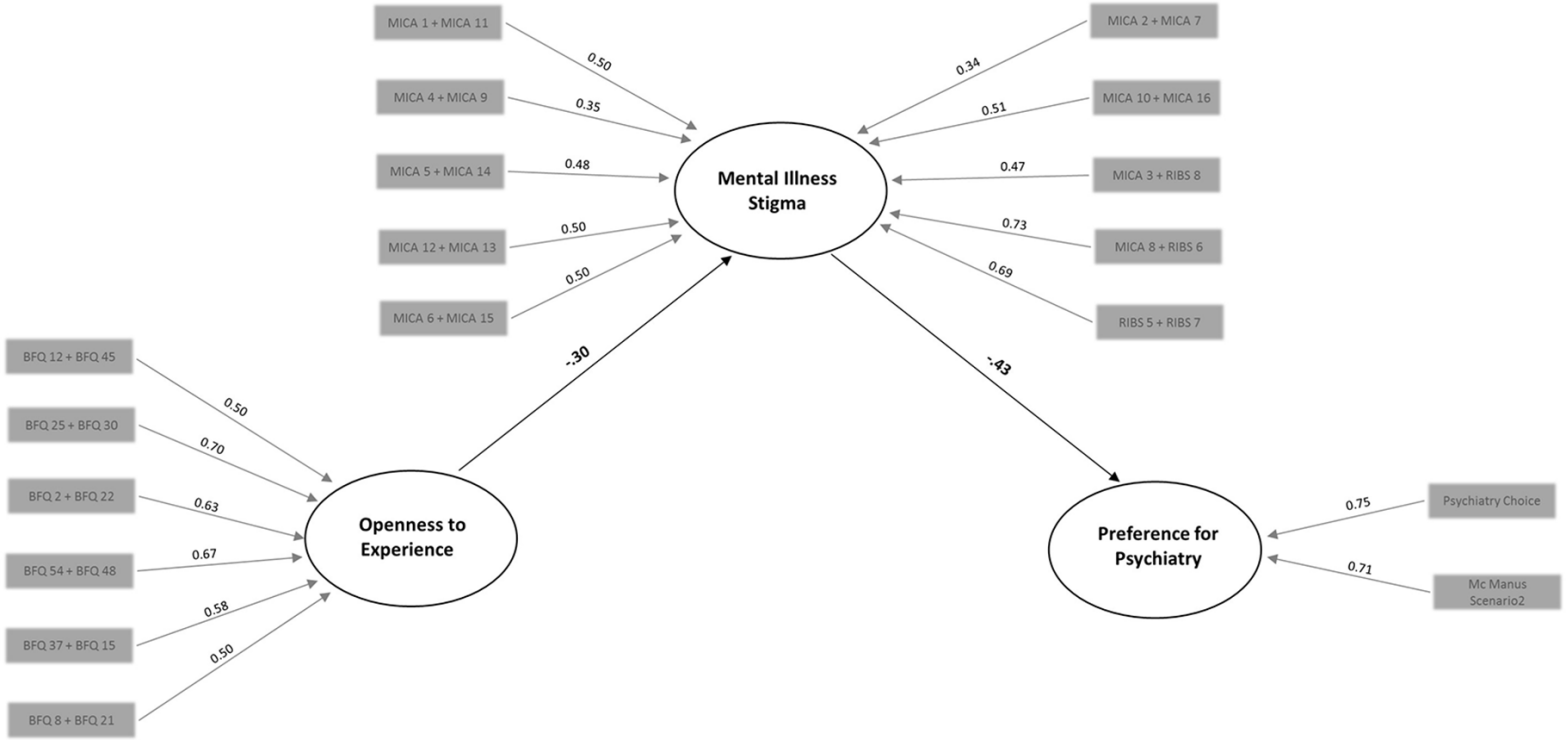
The mediation model of mental illness stigma on the relationship between openness and preference for psychiatry. Statistically significant (*p* < 0.01) coefficients are shown in bold. Parcels used for the measurement of latent variables together with loading coefficients considered in this investigation are represented by the gray boxes.

Descriptive statistics were computed, and Student’s *t* tests were performed to examine gender differences. Associations between the preference for psychiatry, openness to experience, and mental illness stigma were assessed by Pearson *r* coefficients, utilizing SPSS 25 software. To test the mediation effect of mental illness stigma on the relationship between openness to experience and preference for psychiatry, a structural equation modeling (SEM) approach was performed using MPLUS 7.1 software with bootstrapped (*N* = 5,000) ML estimation methods (46). Following recent recommendations (47, 48), we opted for an SEM approach to investigate mediation, since it compares more favorably than the usual regression-based approaches to mediation (49). In the construction of the latent variables of the models, openness to experience was estimated by 6 parcels based on item-total correlations (50), mental illness stigma was estimated by 10 parcels calculated from items of MICA-2 and RIBS, whereas “preference for psychiatry” was estimated by two single items (see **Figure 2**). We specified the basic model and hypothesized “full mediation” (49), meaning that no direct effect of openness to experience on preference for psychiatry would remain once indirect effects were considered (i.e., mediated by mental illness stigma). The fit of the full-mediation model was compared with the fit of the “partial mediation” model (in which direct and indirect paths of openness to experience on preference for psychiatry were both included) by computing a scaled nested *χ* ^2^ difference test (51). The MPLUS 7.1 software provides a series of indices of the model’s fit, including the Tucker–Lewis index and comparative fit index (52), with >0.90 being considered an adequate data–model adaptation (53), the root mean square residual, and the root mean square error of approximation index with <0.08 representing an acceptable one and <0.06 indicating a good data–model fit (54, 55).

## Results

### Individual Traits and Mental Illness Stigma

The main descriptive characteristics of our sample are presented in **Table 1**. Compared to male students, female students obtained higher scores in conscientiousness (*p* < 0.001) and lower scores in emotional stability (*p* = 0.002). A higher level of mental illness stigma was reported by male students compared to female students (*p* < 0.001), while no significant differences were found in terms of avoidant behavior and/or social distance (see **Table 1**). Furthermore, gender differences were observed regarding fear of specific mental illnesses, with female students reporting higher levels of fear of eating disorders compared to students (*p* = 0.002).

Bivariate correlations performed to assess the association between personality traits and mental illness stigma revealed that both the cognitive (MICA-2 scale) and behavioral components (RIBS scale) of mental illness stigma were significantly and negatively correlated with the openness to experience trait and the agreeableness trait (see **Table 2**). We also found a negative correlation between the consciousness trait and MICA-2. As was expected, fear of mental illness was significantly and negatively related with neuroticism and the emotional stability trait. Of note, the cognitive and behavioral components of mental illness stigma as measured by the MICA-2 and RIBS scales, respectively, were highly correlated, whereas the emotional aspect of stigma as measured by fear of mental illness was only positively correlated with the behavioral component of avoidance and social distance measured by RIBS (see **Table 2**).

**Table 2:** Zero order correlations between Big Five traits, Mental Illness Clinicians’ Attitude scale (MICA-2), Reported and Intended Behavior Scale (RIBS), fear of mental illness, and preference for psychiatry.

	Measures	1	2	3	4	5	6	7	8	9
1	Openness to experience	1								
2	Consciensciousness	0.183**	1							
3	Energy	0.326**	0.250**	1						
4	Agreableness	0.408**	0.215**	0.369**	1					
5	Neuroticism	0.233**	0.089	0.292**	0.332**	1				
6	MICA-2	−0.262**	−0.137*	−0.044	−0.346**	−0.003	1			
7	RIBS	−0.136*	0.037	−0.017	−0.239**	0.013	0.525**	1		
8	Fear of mental illness	−0.028	−0.050	−0.046	0.006	−0.251**	0.068	0.142*	1	
9	Preference for psychiatry	0.135*	−0.061	−0.044	0.024	−0.101	−0.253**	−0.235**	0.036	1

*The correlation is significant at level 0.05 (two tailed).

**The correlation is significant at level 0.01 (two tailed).

### Preference for Psychiatry Specialty, Personality Traits, and Mental Illness Stigma

While psychiatry is not a first choice compared to other medical specialties (see **Figure 1**), male and female students reported similar scores on preference for psychiatry as a future medical career (see **Table 1**). As shown in **Table 2**, a preference for psychiatry was positively correlated with openness to experience trait and negatively correlated with both MICA-2 and RIBS. No significant relation was found between preference for psychiatry and other personality traits or fear of mental illness (all *p*s > 0.05).

### Mediation Effect of Mental Illness Stigma on the Relationship Between Openness to Experience and Preference for Psychiatry Specialty

A statistical comparison between the partial- and full-mediation models showed that including direct effect of openness to experience on preference for psychiatry specialty (i.e., the partial mediation model) did not significantly improve the fit of the model, as indicated by the nonsignificant difference of the scaled nested *χ* ^2^ difference test: Δ *χ* ^2^ = 0.30, *p* = 0.58. Hence, the full-mediation model was retained due to greater parsimony. The full-mediation model (see **Figure 2**) provided adequate indices according to recommended cut-off values (51, 54): root mean square residual = 0.06, root mean square error of approximation = 0.05 (with 90% CI limits of 0.035 and 0.059); comparative fit index = 0.91, Tucker–Lewis index = 0.90 and explained 18% of the variance. In the model, mental illness stigma was found to be strongly and negatively associated with both openness to experience (*β* = −0.30, *p* < 0.01) and preference for psychiatry (*β* = −0.43, *p* < 0.01). Regarding the mediation, results showed a positive and significant effect (*β* = .13; *p* = 0.01) of mental illness stigma on the relationship between openness to experience and preference for psychiatry. The significance of this mediation effect was confirmed after bootstrapping procedure (with 99% CI limits of 0.04 and 0.25).

## Discussion

The present study evaluated the impact of mental illness stigma in mediating the effects of openness to experience on preference for psychiatry specialty as a career choice in a sample of second-year medical students. Importantly, we found that the stigma attached to mental illness impinges on the preference for psychiatry, fully accounting for the effect of the openness to experience trait, a trait that has been traditionally linked with preferences for psychiatry. To our knowledge, this is the first evidence highlighting the role of mental illness stigma in limiting the effects of personality traits on the preference for psychiatry as a future medical career choice.

Our results show that psychiatry is not among the first-choice specialties for future medical careers, which is in line with research on similar samples in European countries (56). Furthermore, our prediction regarding the relationship between preference for psychiatry, personality traits, and mental illness stigma was only partially confirmed: in line with research in this domain (4, 5, 10–14), a preference for the psychiatry specialty was found to be significantly and positively associated with the openness to experience trait. However, the size of the correlation was small, and no other significant correlation was found for any of the other Big Five factors, indicating that personality traits do not play a determinant role in the choice of psychiatry as future career option among second-year medical students. On the other hand, our data emphasized the role of mental illness stigma, which was showed by quite a strong negative association with preference for psychiatry of both MICA-2 and RIBS scales.

Our data showed no significant gender differences in preference for the psychiatry specialty, which is in line with previous studies (56–58), but see (12) for different results. MICA-2 levels were higher in male than in female individuals, but no significant gender differences were reported for RIBS scale nor for the fear of specific mental illnesses, except for the fear of eating disorders, which was in line with literature showing a predominantly female incidence (59, 60). Congruent with research demonstrating a reliable link between certain personality traits and levels of prejudice (16–18), both MICA-2 and RIBS were negatively associated with openness to experience and agreeableness traits.

With respect to the main aim of the study, one of the strengths of our results is their demonstrating that the association between openness to experience and preference for psychiatry is fully mediated by mental illness stigma. As evidenced by the full-mediation model’s results, stigmatizing attitudes and behaviors about persons affected by mental illness have an impact on the preference for psychiatry as a career choice, an impact that goes above and beyond the openness to experience trait. To the best of our knowledge, this is the first evidence revealing the determinant interaction that exists between personality traits and attitudes about mental illness when it comes to choosing a career in psychiatry. Important implications can be drawn from these findings in terms of awareness raising and prompting medical students to question their own possibly stigmatizing attitudes toward people with mental illness, especially at a very early stage of their formation. From an educational perspective, it would be useful to include our findings in targeted seminars on medical career choice. Such seminars could not only facilitate informed decision making regarding possible specialties but also contribute to reducing stigmatizing attitudes toward people with mental illness, whom students might encounter in their future careers. On a more applicative note, it is of crucial importance to strengthen the work on stigma awareness and reduction in medical students as early as possible in their educational programs. In particular, scenario-based learning in different settings, simulating primary care, or psychiatric units might be useful in preparing medical students for a potential future in psychiatry while identifying and addressing implicit stigmatizing attitudes toward mental illness and/or psychiatry as a medical discipline (61, 62).

The present study is not exempt of limitations. First, our sample was composed of second-year medical students at the early stages of their education training, and the convenience sampling and the cross-sectional design might limit the interpretation of our results. Second, the fact that we used a MICA-2 scale and an *ad hoc* questionnaire on fear of mental illness that have not yet been validated in Italian might represent another limitation. In addition, this was a country-specific study, and it is possible that our findings reflect contextual characteristics of the Italian sociocultural and educational setting that could have had a role into shaping medical students’ attitudes toward mental illness and consequently their preference for psychiatry as a specialty (57). Future studies employing a longitudinal design are needed to assess changes in students’ attitudes toward patients with mental illness and/or toward psychiatry as a specialty throughout their medical educations and as a result of specific curricular or stigma reduction programs (e.g., special psychiatric modules or clinical placement in psychiatric residency programs, antistigma interventions) (36, 63, 64). Future research should also examine the relationship between personality traits and mental illness stigma in mental health professionals.

Notwithstanding these limitations, the present data reveal the role of mental illness stigma in limiting effects of openness to experience on students’ preferences for psychiatry as a specialty choice, importantly highlighting the need to introduce stigma awareness and stigma reduction programs as early as possible in innovative medical education curricula.

## Data Availability Statement

The datasets generated for this study are available on request to the corresponding author.

## Ethics Statement

This study was carried out in accordance with national and institutional guidelines. Ethical approval of the study protocol was granted by the Institutional Review Board of University of Bologna and all participants provided written informed consent in accordance with the Declaration of Helsinki. The request for consent to participate was made through verbal and written communications which comprehensively explained the nature and purpose of the study. Participation was anonymized and each participant was attributed a code.

## Author Contributions

EF, VC, and PR contributed to the conception and design of the study. AA, MM and EC contributed to the acquisition of the data. EC and PR contributed to data analysis and interpretation of the data. EF and PR drafted the manuscript. EF, AA, MM, VC, EC and PR were involved in the critical revision of the manuscript.

## Funding

RFO funds awarded to AA will support the coverage of article publication fees.

## Conflict of Interest

The authors declare that the research was conducted in the absence of any commercial or financial relationships that could be construed as a potential conflict of interest.
